# Modern classification and molecular-genetic aspects of osteogenesis imperfecta

**DOI:** 10.18699/VJ20.614

**Published:** 2020-03

**Authors:** A.R. Zaripova, R.I. Khusainova

**Affiliations:** Institute of Biochemistry and Genetics – Subdivision of the Ufa Federal Research Centre of the Russian Academy of Sciences, Ufa, Russia; Institute of Biochemistry and Genetics – Subdivision of the Ufa Federal Research Centre of the Russian Academy of Sciences, Ufa, Russia Republican Medical-Genetic Center, Ufa, Russia

**Keywords:** osteogenesis imperfecta, collagen, bone fragility, bisphosphonates, multiple fractures, незавершенный (несовершенный) остеогенез, коллаген, хрупкость костей, бисфосфонаты, множественные переломы

## Abstract

Osteogenesis imperfecta (imperfect osteogenesis in the Russian literature) is the most common hereditary form of bone fragility, it is a genetically and clinically heterogeneous disease with a wide range of clinical severity, often leading to disability from early childhood. It is based on genetic disorders leading to a violation of the structure of bone tissue, which leads to frequent fractures, impaired growth and posture, with the development of characteristic disabling bone deformities and associated problems, including respiratory, neurological, cardiac, renal impairment, hearing loss. Osteogenesis imperfecta occurs in both men and women, the disease is inherited in both autosomal dominant and autosomal recessive types, there are sporadic cases of the disease due to de novo mutations, as well as X-linked forms. The term “osteogenesis imperfecta” was coined by W. Vrolick in the 1840s. The first classification of the disease was made in 1979 and has been repeatedly reviewed due to the identification of the molecular cause of the disease and the discovery of new mechanisms for the development of osteogenesis imperfecta. In the early 1980s, mutations in two genes of collagen type I (COL1A1 and COL1A2) were first associated with an autosomal dominant inheritance type of osteogenesis imperfecta. Since then, 18 more genes have been identified whose products are involved in the formation and mineralization of bone tissue. The degree of genetic heterogeneity of the disease has not yet been determined, researchers continue to identify new genes involved in its pathogenesis, the number of which has reached 20. In the last decade, it has become known that autosomal recessive, autosomal dominant and X-linked mutations in a wide range of genes, encoding proteins that are involved in the synthesis of type I collagen, its processing, secretion and post-translational modification, as well as in proteins that regulate the differentiation and activity of bone-forming cells, cause imperfect osteogenesis. A large number of causative genes complicated the classical classification of the disease and, due to new advances in the molecular basis of the disease, the classification of the disease is constantly being improved. In this review, we systematized and summarized information on the results of studies in the field of clinical and genetic aspects of osteogenesis imperfecta and reflected the current state of the classification criteria for diagnosing the disease.

## Abstract (ru)

Незавершенный остеогенез (несовершенный остеогенез в русскоязычной литературе) – наиболее распространенная наследственная форма ломкости костей, генетически и клинически гетерогенное заболевание с широким спектром клинической тяжести, основное клиническое проявление которого – множественные переломы начиная с натального периода жизни, зачастую приводящие к инвалидизации с детского возраста. К основным клиническим признакам незавершенного остеогенеза относятся голубые склеры, потеря слуха, аномалия дентина, повышенная ломкость костей, нарушение роста и осанки с развитием характерных инвалидизирующих деформаций костей и сопутствующих проблем, включающих дыхательные, неврологические, сердечные, почечные нарушения. Незавершенный остеогенез встречается и у мужчин, и у женщин, заболевание наследуется как по аутосомно-доминантному, так и аутосомно-рецессивному типам, существуют спорадические случаи заболевания, обусловленные мутациями de novo, а также обнаружены Х-сцепленные формы. Термин «незавершенный остеогенез» был введен W. Vrolick в 1840-х гг. Первая классификация заболевания сделана в 1979 г. и неоднократно пересматривалась из-за идентификации молекулярной причины заболевания и открытия новых механизмов развития незавершенного остеогенеза. В начале 1980-х гг. мутации в двух генах коллагена типа I (COL1A1 и COL1A2) впервые были ассоциированы с аутосомно-доминантным типом наследования незавершенного остеогенеза. С тех пор идентифицированы еще 18 генов, продукты которых участвуют в процессах формирования и минерализации костной ткани. До сих пор не определена степень генетической гетерогенности заболевания, исследователи продолжают идентифицировать новые гены, вовлеченные в его патогенез, число которых достигло 20. В последнее десятилетие стало известно, что аутосомно-рецессивные, аутосомно-доминантные и Х-связанные мутации в широком спектре генов, кодирующих белки, участвующие в синтезе коллагена типа I, его процессинге, секреции и посттрансляционной модификации, а также в белках, регулирующих дифференцировку и активность костеобразующих клеток, вызывают несовершенный остеогенез. Большое количество причинных генов усложнило классическую классификацию заболевания, и в связи с новыми достижениями в области молекулярных основ незавершенного остеогенеза постоянно совершенствуется и классификация. В этом обзоре мы систематизировали и обобщили информацию о результатах исследований в области изучения клинико-генетических аспектов незавершенного остеогенеза и отразили современное состояние классификационных критериев диагностики заболевания.

## Introduction

Osteogenesis imperfecta (OI), also known as brittle bone disease, is a clinically and genetically heterogeneous hereditary disease of connective tissue, the main cause of which is a genetically determined violation of the quality of bone tissue, leading to frequent fractures with the development of disabling bone deformities and a complex of concomitant problems on the part of the respiratory, cardiovascular, neuromuscular systems.

Worldwide osteogenesis imperfecta occur with a frequency approximately of 1 in every 30,000 births. The desease affects both men and women. In Russia osteogenesis imperfecta is the most common genetic bone disease – one case per 10– 20 thousand newborns. According to the ministry of health in 2014 in Russia there are 556 adults and children with osteogenesis imperfecta (Kruchkova, Kruglov, 2014). In the past decade, (mostly) recessive, dominant and X-linked defects in a wide variety of genes encoding proteins involved in type I collagen synthesis, processing, secretion and post-translational modification, as well as in proteins that regulate the differentiation and activity of bone-forming cells have been shown to cause osteogenesis imperfecta (Marini et al., 2017). Also sporadic cases of osteogenesis imperfecta are affected by de novo mutations, which frequency is necessary to find out.

Nowadays, 20 genes are responsible for the development of different types of osteogenesis imperfecta and the search of new genes that take part in pathogenesis of the disease is still continuing. In past five years 6 new genes, which take part in pathogenesis of osteogenesis imperfecta, were identified. The last gene was identified in 2018 and it is not still known if the disease clinically and genetically heterogenious. Genetic defects, that lead to OI, are transformed into the defects of collagen synthesis, structures of its chains, post-translational modification of collagen, proper twisting into a triple helix and stitching (Nadyrshina et al., 2012). Also there are defects of bone tissue mineralization and osteoblasts differentiation. Due to the identification of new molecular causes of the disease, continuous improvement of diagnostic criteria and revision of classification of OI is carried out.

The aim of this article is the review of current state of clinical and genetic aspects of OI and the generalization of the results of molecular pathogenesis of the disease.

## Evolution of classification criteria of osteogenesis imperfecta

The existance of clinical features, which are corresponded to osteogenesis imperfecta, had been known from ancient times. The earliest case of the disease was identified in 1000 BC in the study of a partially mummified skeleton of an infant from ancient Egypt (Lowenstein, 2009; Ramachandran, Jones, 2018). Also exist a story about Ivar The Boneless was a Viking leader who invaded Anglo-Saxon England. According to the Tale of Ragnar Lodbrok, Ivar’s bonelessnes was the result of a curse. He was born with weak bones. While the sagas describe Ivar’s physical disability, they also emphase his wisdom, cunning, and mastery of strategy and tactics in battle (Mahoney, 2017). Different publications of brittle bones and hearing loss studies have been appearing in medical literature since 1600. J.F. Lobstein и W. Vrolik were one of the first people, who could get the etiology of osteogenesis imperfecta. In 1825 J.F. Lobstein got some information about 3 sick children of different age. They had fractures of tubular bones without any reason. Author decided to name this disease as “ostepsathyrosys” and in his treatise on pathological anatomy devoted an entire chapter of it.

In 1849 W. Vrolik described “Osteogenesis Imperfecta” as syndrome of brittle bone with a lot of fractures which happened in prenatal period or immediately after birth. Searching the literature, we can see how gradually congenital bone fragility stood out from the concept of rickets. Since 1900 the authors began to point out the genetic nature of osteogenesis imperfecta. 

J. Spurway in 1896 reported the first instance in which the condition of fragile bones (fragilitas ossium) was associated with blue scleras. E. Bronson in 1917 and J. Hoeve and A. Kleyn in 1918 added to syndrome the third feature, deafness. J.A. Key in 1926 reffered to the syndrome as “hereditary hypoplasia of the mesenchyme” and called attention to the hypotonicity of the ligaments with hypermobile joints. The first classification of osteogenesis imperfecta was made by E. Looser, in 1906 who divided the condition on two forms, osteogenesis imperfecta congenita (also known as Vrolik disease) and osteogenesis imperfecta tarda (also known as Ekman-Lobstein disease) to distinguish the early and late forms of the disease.

In the 1970s, Dr. D. Sillence and his team of researchers in Australia developed the system of categorization using “Types” that is currently in use. His original four classifications (Type I, Type II, Type III and Type IV) combine clinical symptoms with genetic components. This listing is based on the number of people in the study who had similar symptoms. The types do not go from mildest to most severe. This classification system has been generally accepted world wide since 1979 OI continues to evolve as new information is discovered (Yakhyayeva et al., 2015b). Later this classification was supplemented by M. Ramachandran et al. (Pigarova et al., 2017), which also took into account the violation of dentinogenesis, OI the IV type of OI was subdivided into subtype B, which is accompanied by defects of dentinogenesis, and subtype A, which does not have these violations.

In 2000, F.H. Glorieux presented a classification of osteogenesis imperfecta, in which, in addition to the already known types, four more types of OI (V, VI, VII, VIII) were identified that are not associated with the pathology of type I collagen. In this classification, modern advances in the field of molecular genetic studies of the disease were taken into account (Table 1).

**Table 1.**
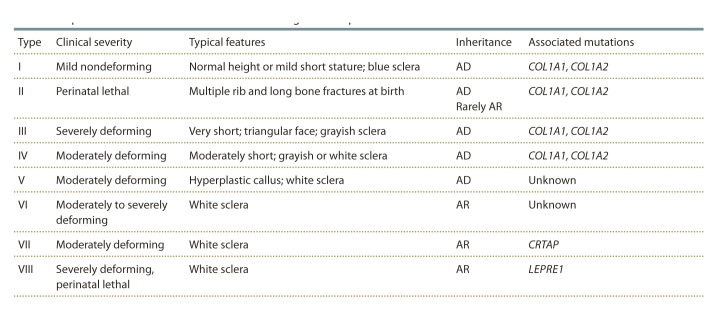
Expanded F.H. Glorieux classification of osteogenesis imperfecta Note. AD – autosomal dominance; AR – autosomal recessivity; COL1A1 and COL1A2 – genes encoding type I collagen; CRTAP – cartilage-associated protein; LEPRE1, also known as P3H1, prolyl-3-hydroxylase 1.

In less than 5 % of patients diagnosed with OI, type V occurs, which is inherited in an autosomal dominant type. The clinical phenotype of OI type V differs from other types of OI and is characterized by calcification of the interosseous membrane of the forearm and the formation of hyperplastic callus. OI–V has a wide spectrum of disease severity.

Type VI OI is clinically similar to types II and IV but has different characteristic histological picture – forming osteoid due to a violation of mineralization (Glorieux et al., 2002).

Type VII is manifested by deformations of long bones, shortening of the proximal limbs, coxa vara (varus deformities of the femoral neck), accompanied by normal dentinogenesis and the usual color of the sclera. It is characterized by an autosomal recessive type of inheritance. VII type OI is caused by a gene mutation in the chromosome 3p22-24.1, which encodes a protein associated with cartilage (CRTAP). CRTAP is a co-factor for post-translational modification of type I collagen. The severity of the disease depends on the degree of CRTAP deficiency. In the complete absence of CRTAP protein, prenatal death occurs, or the baby is born with severe OI (Ward et al., 2002).

Type VIII– a severe type of the desease, clinically similar to type II of OI, characterized by an autosomal recessive type of inheritance, associated with the mutation in LEPRE1. Diagnosed at perinatal age. Severe bone deformities, white sclera, are characteristic, accompanied by normal dentinogenesis (Fratzl-Zelman et al., 2016).

Types I–V are predominantly autosomal dominant inheritance, VI–XVIII are autosomal recessive. When new genes were discovered, the classification expanded, and by 2015, the number of forms of the disease reached 18.

Osteogenesis imperfecta type I is characterized by the presence of a defect in the COL1A1 gene, which leads to a decrease in the amount of type I collagen produced; in types II–IV, due to mutations in the COL1A1 and COL1A2 genes, type V is due to mutations in the IFITM5 gene and dysregulation of bone mineralization, type VI occurs due to a mutation in the SERPINF1 gene, which leads to a defect in bone mineralization; types VII (CRTAP gene), VIII (LEPRE1 gene, also known as P3H1) and IX (PPIB gene) are the result of a defect in the collagen 3-hydroxylation process. The cause of osteogenesis imperfecta of the X and XI types is a violation of the processing and cross-linking of collagen due to mutations in the SERPINH1 and FKBP10 genes, respectively. Mutations in the PLOD2 and BMP1 genes lead to incomplete type XII osteogenesis. These genes are involved in post-translational modification, processing, folding, secretion, and crosslinking of type I procollagen. Types XIII–XVIII of osteogenesis imperfecta are characterized by a violation of the differentiation of osteoblasts: mutations in the SP7 gene lead to the manifestation of the XIII type, in the TMEM38B gene – the XIV type, in WNT1 – the XV type, in CREB3L1 – the XVI type, in SPARC – the XVII type, in MBTPS2 – XVIII type (Marini et al., 2017).

The classification of the disease, taking into account the molecular pathogenesis of the disease, complicated the work of clinical doctors and in 2016 the International committee of nomenclature of constitutional disorders of the skeleton, INCDS) reduced the classification to 5 forms, retaining 4 types, which were originally described by silence and adding a 5th type. In total, 5 groups of the disease were identified using the Arabic digital system, which indicates the unifying phenotypic characteristics, and individual (characteristic for a particular type) changes still retained their original Roman designation (Table 2) (Ignatovich et al., 2018). This characteristic leaves room for the inclusion of new genes found as the cause of osteogenesis imperfecta until the degree of heterogeneity of the disease is identified.

**Table 2.**
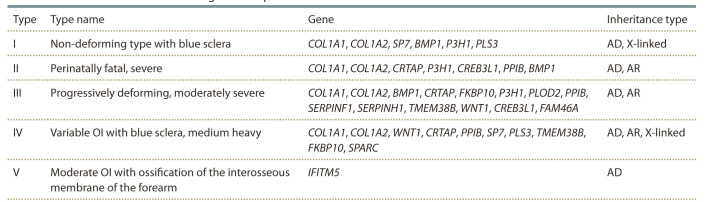
Modern classification of osteogenesis imperfecta Note. AD is an autosomal dominant type of inheritance; AR is an autosomal recessive type of inheritance.

Thus, the classification of osteogenesis imperfecta has undergone a number of fundamental changes associated with advances in the study of the molecular pathogenesis of the disease. The degree of heterogeneity of the disease has not yet been determined, the incidence of de novo cases has not been estimated, and therefore, it will probably continue to improve the classification criteria for the diagnosis of osteogenesis imperfecta.

## Modern views on the etiology and pathogenesis of osteogenesis imperfecta

Osteogenesis imperfecta is characterized by wide clinical and genetic heterogeneity; earlier, the disease was referred to as collagenopathies, because in most cases, the structure and function of the main protein of bone tissue – type I collagen, as well as its stability are disturbed. Later, in patients with osteogenesis imperfecta, mutations were revealed in genes that do not participate in the formation of collagen structure and folding (Tournis, Dede, 2017).

To date, 20 genes responsible for the development of OI have been identified. The autosomal dominant type of ND inheritance in most cases is caused by defects in the COLIA1 or COLIA2 genes of type I collagen chains encoding α1 (I) and α2 (I) peptide type I collagen chains, respectively (Ignato-vich et al., 2018). Autosomal dominant disease inheritance options have also been described in several patients with mutations in the IFITM5 (MIM: 614757) and P4HB (MIM: 176790) genes.

P4HB encodes the beta subunit of prolyl 4-hydroxylase, which is involved in prolyl hydroxylation and folding of procollagen (Li et al., 2019), and IFITM5 is a gene specific for osteoblasts associated with matrix mineralization (Glorieux et al., 2000). The IFITM5 gene is located on chromosome 11 (p15.5) in a cluster of related genes (IFITM1, 2, 3, and 10) and belongs to the family of genes encoding proteins containing two transmembrane domains that perform various significant cellular functions (Yakhyayeva et al., 2014).

Osteogenesis imperfecta is also transmitted in an autosomal recessive manner of inheritance, which is caused by mutations in the following genes: BMP1 (MIM: 112264) (Asharani et al., 2012), CRTAP (MIM: 605497) (Morello et al., 2006), FKBP10 (MIM: 607063) (Barnes et al., 2012), P3H1 (MIM: 610339) (Cabral et al., 2007), PLOD2 (MIM: 601865) (Puig-Hervás et al., 2012), PPIB (MIM: 123841) (VanDijk et al., 2009), SEC24D (MIM: 607186) (Zhang et al., 2017), SERPINH1 (MIM: 600943) (Christiansen et al., 2010) and TMEM38B (MIM: 611236) (Rubinato et al., 2014), which are involved in post-translational modifications, processing, coagulation, secretion and cross-linking of procollagen (I). However, there is another group of OI loci with AR type of inheritance, which are not recognized as directly involved in the biosynthesis of type I collagen, but play a role in the mineralization or development of osteoblasts. This second group of genes includes CREB3L1 (MIM: 616215) (Symoens et al., 2013), SERPINF1 (MIM: 172860) (Becker et al., 2011), SP7 (MIM: 606633) (Lapunzina et al., 2010), SPARC (MIM: 182120) (Mendoza- Londono et al., 2015) and WNT1 (MIM: 164820) (Laine et al., 2013; Pyott et al., 2013). Finally, mutations in the PLS3 (MIM: 300131) genes (Costantini et al., 2018) and MBTPS2 (MIM: 300294) were associated with two different forms of X-linked forms of OI.

It is known that there are two genes that encode proteins that are part of the metabolic chain that regulate intramembrane proteolysis (RIP) in osteoblasts, leading to the formation of the phenotype of osteogenesis imperfecta. During the intramembrane proteolysis, endopeptidases S1P (encoded by the MBTPS1 gene) and S2P (encoded by the MBTPS2 gene) in the Golgi membrane sequentially cleave regulatory proteins transported from the endoplasmic reticulum during stress endoplasmic reticulum or sterol metabolite deficiency. In patients with mutations in the MBTPS2 gene, lysine hydroxylation of the α1 (I) chain and α2 (I) chain is reduced, collagen crosslinking is altered, and bone tissue strength is impaired. One of the transcription factors activated by RIP is a specific astrocyte-induced substance (OASIS; encoded by CREB3L1). A deficiency of this substance has been reported in association with a family with severe osteogenesis imperfecta. OASIS is a stress transducer of the endoplasmic reticulum, which regulates the transcription of genes involved in the development, differentiation and maturation of osteoblasts. In mice with the knocked out CREB3L1 gene, severe osteopenia was observed with spontaneous fractures and a decrease in the production of type I collagen in the bone (Lindert et al., 2016).

In 2018, another gene was discovered – FAM46, which also leads to osteogenesis imperfecta with an autosomal recessive type of inheritance. FAM46A is a member of the superfamily of nucleotidyl transferase folded proteins, but its exact function is currently unknown. However, there is some evidence pointing to the corresponding role of FAM46A in bone de-velopment. Using RT-PCR analysis, specific FAM46A expression was detected in human osteoblasts and, interestingly, a nonsense mutation in FAM46A was recently discovered in a mouse model derived from ENU (N-ethyl-N-nitrosourea), characterized by a decrease in body length, limbs, deformation of the ribs, pelvis and skull and a decrease in the thickness of the cortex in long bones (Doyard et al., 2018) (Table 3).

**Table 3.**
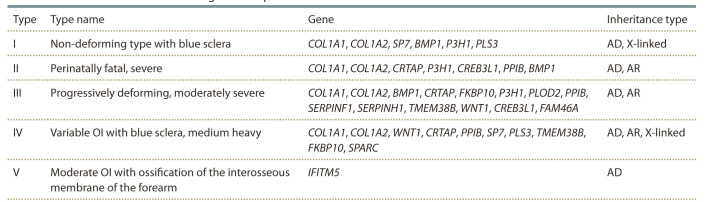
Characterization of genes and their protein products responsible for the development of OI

About 90 % of the 3,000 people from the incomplete osteogenesis database (http://www.le.ac.uk/ge/collagen/) have changes in either the COL1A1 gene or COL1A2, and the remaining 10 % show homozygous or heterozygous mutations in other genes involved in the pathogenesis of OI. However, major sequencing centers that offer a panel of causal mutations associated with incomplete osteogenesis identify a lower frequency of structural mutations in the COL1A1 and COL1A2 genes in patients with a moderate to severe clinical presentation of the disease. For example, heterozygous mutations in the COL1A1 or COL1A2 genes were identified in 77 % of 598 patients with ND from the Shreiners Clinic (Montreal, Canada), 9 % had one mutation in the IFITM5 gene, and the rest had homozygous or heterozygous mutations in other genes causing incomplete osteogenesis. Lethal mutations in the collagen gene could be lost in this study. In populations with a high level of blood relationship, the frequency of incomplete osteogenesis is higher, for example, among African Americans in the United States of America, the frequency of the mutant variant in the P3H1 gene (previously called LEPRE1 encoding shedding 3-hydroxylase 1) is about 1 in 240 people. Homozygosity for this so-called West African allele accounts for 25 % of all cases of incomplete lethal osteogenesis in this population, which may be clinically erroneously classified as type II OI. Among the West Africans of Ghana and Nigeria, the frequency of occurrence of this allele is 1.5 %, which can lead to a frequency of lethal recessive incomplete osteogenesis equal to the frequency of de novo mutations in type I collagen.

Despite the large number of mutations recorded in the database on incomplete osteogenesis (https://oi.gene.le.ac.uk), each population has its own spectrum consisting of a small number of mutations, with each researcher finding previously undescribed in mutation literature.

As in the case of other recessive diseases, in some populations there are isolated cases of mutations in rare genes that are not found in other populations: an exon deletion in the TMEM38B gene was found in a family from Saudi Arabia; reading frame offset in the FKBP10 gene was found in patients from Turkey; missense mutations in the WNT1 gene in the Hmong ethnic group from Vietnam and China (Marini et al., 2017). Among the population of northern Ontario (Canada), the intron variant destabilizes the mRNA of the CRTAP gene (which encodes a protein associated with cartilage) and develops the phenotype of incomplete osteogenesis type VII.

The clinical picture of OI and the severity of the disease are diverse, they can be manifested by lethal variants, obvious abnormalities of the skeleton in children, or have an easy manifestation in people of mature age. The severity of the disease is determined by the frequency of fractures, progressive deformity, chronic bone pain and loss of mobility. Due to the clinical heterogeneity of the disease, there are difficulties in diagnosing and verifying the diagnosis; in children with OI, a delay in physical development, scoliosis, progressive deformations of long bones, hearing loss, pathology of teething are revealed, therefore only the identification of the molecular cause of the disease allows an accurate diagnosis to be established.

Thus, significant progress has been made in the study of the molecular pathogenesis of incomplete osteogenesis, but the degree of heterogeneity of the disease remains to be determined. With the development of genotyping technologies and the widespread adoption of deep sequencing and full-exomic sequencing methods, it has become possible to identify not only new mutations in known genes, but also to identify new genes involved in the development of the disease.

## Prospects for the treatment of incomplete osteogenesis

Currently, active research is being conducted on the possibilities of targeted therapy for patients with hereditary diseases, taking into account the molecular defect. Encouraging results were obtained with the pathogenetic treatment of cystic fibrosis.

Bisphosphonates (BP) are the main drug for the treatment of both children and adult patients with OI. It is believed that BP can be less effective or even lead to adverse consequences in cases of insufficient calcium intake and/or vitamin D deficiency (Weaver et al., 2016).

There are also preclinical and a small number of clinical studies in adult patients with OI regarding denosumab, a monoclonal antibody targeted at RANKL (receptor activator of the nuclear factor kappa-B ligand). Regarding anabolic therapy, teriparatide, currently the only available anabolic agent, has shown promising results in adult patients with type I OI. Preclinical studies show that inhibition of TGF-beta signaling, as well as inhibition of sclerostin, can also play a role in treating bone fragility. In addition to pharmacological interventions, the multidisciplinary approach provided by experienced orthopedic surgeons, dental care specialists, physiotherapists and kinesitherapy specialists is of paramount importance for providing the best possible medical care.

Currently, bisphosphonates are widely used to treat children with OI. It has been shown that both oral (alendronate, risedronate) and intravenous administration of BP (pamidronate, zoledronate, neridronate) improve the level of BMD, especially in the spine. However, data from a randomized, placebo-controlled trial regarding fracture response, pain relief, and motor activity improvement are still missing. Recent studies have not found a consistent decrease in the frequency of fractures and an improvement in the clinical status of patients in the treatment of BP (Dwan et al., 2014).

Concerning the effect of BF in adults with OI, there is limited evidence that tested the effect of various BFs on BMD levels. Almost all studies reported a beneficial effect on the level of BMD of the lower spine (an increase of up to 13.9 %) with less pronounced effects on the total level of BMD of the thigh (an increase of up to 4.3 %) (Lindahl et al., 2014). Recently, a number of reports have been published about atypical hip fractures in adult patients with OI receiving treatment with BF.

A number of studies have evaluated the effect of denosumab in patients with OI caused by a mutation in SERPINF1, characterized by a weak response to BP, as well as in patients with OI I/IV (n = 8) and OI III (n = 2) types (Hoyer-Kuhn et al., 2016). The dose used was 1 mg/kg subcutaneously every 3 months. All studies reported a significant increase in BMD and the absence of significant side effects of treatment over a two-year period.

Sclerostin inhibition may be another treatment option for bone fragility in OI. Recently published studies have shown that administering romososumab (a sclerostin-binding monoclonal antibody) within one year reduces the incidence of spinal fractures and osteoporosis in postmenopausal women with osteoporosis (Sinder et al., 2015; Grafe et al., 2016).

In a mouse model with OI, it was shown that increased TGF-β signaling is involved in the OI phenotype, while inhibition of TGF-β improves bone mass and strength. Phase 1 of the study verifies the safety of fresolumumab, a high-affinity neutralizing antibody that targets all 3 TGF-β isoforms, in adults with a mild clinical presentation of OI. Combination therapy with antiresorptive and anabolic agents is another potential treatment option for bone fragility in patients with OI. Other treatments, such as bone marrow transplantation and gene therapy, are in the process of evaluating the effectiveness of treating severe forms of OI (Marini et al., 2017).

Thus, despite the progress made in understanding the pathophysiology of OI, additional research is still needed to determine the best therapeutic approach to this heterogeneous disease.

## Conclusion

Summarizing the foregoing, we can conclude that there has been a breakthrough in the identification of the molecular pathogenesis of incomplete osteogenesis, which is due to the introduction of modern next-generation sequencing technologies (NGS). However, questions about the prevalence of the disease as a whole and its individual clinical forms in various world populations are still far from over. Also, a final determination of the degree of molecular heterogeneity of OI has not been achieved; the identification of new pathogenetic mechanisms of the formation of the phenotype of the disease continues on the basis of identifying new genes involved in the pathogenesis of OI. Currently, attempts are being made to develop targeted therapy for the disease, taking into account new knowledge about the clinical and genetic aspects of OI, but there are still many conflicting results and the solution to the problem of treating the disease is far from complete.

## Conflict of interest

The authors declare no conflict of interest.
